# Bio-Based Polyurethane Foams with Castor Oil Based Multifunctional Polyols for Improved Compressive Properties

**DOI:** 10.3390/polym13040576

**Published:** 2021-02-14

**Authors:** Joo Hyung Lee, Seong Hun Kim, Kyung Wha Oh

**Affiliations:** 1 Department of Organic and Nano Engineering, College of Engineering, Hanyang University, Seoul 04763, Korea; therauss@gmail.com (J.H.L.); kimsh@hanyang.ac.kr (S.H.K.); 2 Department of Fashion, College of Arts, Chung-Ang University, Anseong 17546, Korea

**Keywords:** castor oil, thiol-ene click reaction, multifunctional polyol, polyol blend, polyurethane foam

## Abstract

Currently, most commercial polyols used in the production of polyurethane (PU) foam are derived from petrochemicals. To address concerns relating to environmental pollution, a sustainable resource, namely, castor oil (CO), was used in this study. To improve the production efficiency, sustainability, and compressive strength of PU foam, which is widely used as an impact-absorbing material for protective equipment, PU foam was synthesized with CO-based multifunctional polyols. CO-based polyols with high functionalities were synthesized via a facile thiol-ene click reaction method and their chemical structures were analyzed. Subsequently, a series of polyol blends of castor oil and two kinds of castor oil-based polyols with different hydroxyl values was prepared and the viscosity of the blends was analyzed. Polyurethane foams were fabricated from the polyol blends via a free-rising method. The effects of the composition of the polyol blends on the structural, morphological, mechanical, and thermal properties of the polyurethane foams were investigated. The results demonstrated that the fabrication of polyurethane foams from multifunctional polyol blends is an effective way to improve their compressive properties. We expect these findings to widen the range of applications of bio-based polyurethane foams.

## 1. Introduction

Polyurethane (PU) foams are one of the most prominent classes of polymers that have been widely used in various industrial fields, such as automobiles [1], furniture [2], thermal insulation materials [3], sound insulation materials [4], lightweight structural materials [5], and cushioning materials [6], owing to their advantages such as low cost, low density, and facile fabrication [7]. PU foams, prepared by addition polymerization between multifunctional alcohols and diisocyanates, exhibit a wide range of mechanical properties depending on their reactants. Because the types of commercially available diisocyanates are limited, the polyols act as an important factor in determining the properties of PU foams. Currently, most commercial polyols are derived from petrochemicals; however, concerns about environmental pollution and regulations have led to an interest in using sustainable resources to replace petroleum-based polyols [8]. The most common sustainable bio-based materials are vegetable oils, polysaccharides, cellulose, lignocellulose, and starch [9,10,11,12,13,14,15,16]. Among them, vegetable oils have attracted considerable attention as an alternative to petroleum-based commercial polyols because of their abundance, price competitiveness, low toxicity, inherent bio-degradability, and ease of extraction from bio-renewable resources such as castor [17], soybean [18], canola seeds [19], sunflower [20], grapeseed [21], palm [22], and others [23]. Vegetable oils consist of a triglyceride structure derived from glycerol and three fatty acids. Castor oil (CO) is mainly composed of ricinoleic acid (12–hydroxy–*cis*–9–octadecenoic acid) which is an aliphatic unsaturated chain with a double bond at the C-9 position [24]. It contains a hydroxyl group at the C-12 position in nature, which allows it to be utilized in PU production [25]. However, the successful commercialization of PU produced from CO is limited by the poor mechanical properties of the foam and low productivity, which are attributed to the inherent characteristics of CO, such as low hydroxyl value, low reactivity of secondary OH groups, and steric hindrance [26]. Recently, several studies have focused on overcoming these drawbacks through the modification of castor oil by epoxidation [27], transesterification [28], ozonolysis [29], and radical addition [18].

The thiol-ene click reaction has been attracting considerable attention as a facile and efficient method for producing multifunctional materials [30]. Based on its simple reaction conditions, high rate and yield, and stereo-selectivity, as well as ease of purification, this approach has been considered as a new route to develop vegetable oils as a feedstock for PU synthesis [20,26]. Alagi et al. developed thiol-grafted vegetable oil-based polyols and utilized them to prepare thermoplastic PUs [31]. Feng et al. optimized the reaction conditions for grafting 2-mercaptoethanol onto soybean oil and then applied it to synthesize PU films [18]. Omrani et al. reported a synthetic route for a polyol with a hydroxyl value of 402 mg KOH/g from sunflower oil [20]. We have reported sustainable polyols with gradually increasing functionalities via the thiol-ene click reaction with castor oil [26]. Despite numerous studies, only a few researchers have reported the fabrication of PU foams from multifunctional polyols prepared via thiol-ene click reactions.

In this study, two types of CO-based polyols with different functionalities are prepared using a facile thiol-ene click reaction method. The prepared polyols are blended with CO to attain a series of polyol blends with increasing hydroxyl values. The viscosities of the polyol blends are analyzed. The PU foams derived from CO-based polyol blends were synthesized via a free-rising method. The property changes of the PU foams resulting from the increasing functionality of the polyol blends are thoroughly examined. The structures of the CO-based polyols were analyzed via Fourier transform infrared (FT-IR) spectroscopy and proton nuclear magnetic resonance (^1^H-NMR) spectroscopy. The structural, morphological, thermal, and mechanical properties of the resultant PU foams were characterized via FT-IR spectroscopy, scanning electron microscopy (SEM), the universal testing machine (UTM), and thermogravimetric analysis (TGA).

## 2. Experimental

### 2.1. Materials

CO was purchased from Yakuri Pure Chemical Co., Ltd. (Kyoto, Japan), while 2–Mercaptoethanol and α–thioglycerol were purchased from Tokyo Chemical Industry Co., Ltd. (Tokyo, Japan). Additionally, 2, 2–dimethoxy–2–phenylacetophenon (DMPA), dimethyl cyclohexylamine (DMCHA), and dibutyltin dilaurate (DBTDL) were purchased from Sigma-Aldrich (St. Louis, MI, USA). Ethyl acetate and sodium chloride were purchased from Daejung Chemicals (Siheung, Korea). Polymeric methylene diphenyl diisocyanate (pMDI, Cosmonate M-200, functionality 2.7, NCO index 31%) was supplied by Kumho Mitsui Chemicals, Inc. (Yeosu, Korea). The silicon surfactant (TEGOSTAB B–8462) was supplied by Evonik (Essen, Germany).

### 2.2. Preparation of Castor Oil-Based Polyols

CO-based polyols with high functionalities were prepared by following our previous study with a slight modification for mass production [26]. Briefly, CO, thiols, the photo initiator (DMPA), and ethyl acetate as a solvent were placed in quartz tubes, which were rolled for 24 h in a tube roller placed in a photochemical reactor equipped with ultraviolet lamps. The molar ratio of thiols to carbon–carbon double bonds was set at 4.35:1. After a pre-determined reaction time, the products were collected and washed with distilled water and aqueous sodium chloride solution at least five times. The polyols were dried using MgSO_4_ and the ethyl acetate was removed by rotary evaporation. The final polyol products were dried in vacuo for 24 h. CO-based polyols modified with mercaptoethanol and α-thioglycerol were designated COM and COT, respectively.

### 2.3. Preparation of PU Foams

To fabricate PU foams, prepared polyol, silicon surfactant, catalysts, and deionized water were mixed according to the calculated ratio, followed by stirring at a speed of 4000 rpm for 5 min using a homogenizer. Thereafter, appropriate amounts of pMDI were added to the mixture and the samples were stirred at 10,000 rpm for 10 s. The stirred mixtures were poured into a mold and allowed to rise freely (free-rising method). After completing the reaction, the obtained foams were cured at 80 °C for 3 d. The detailed foam formulation is shown in Table 1. Seven kinds of polyol mixtures were prepared by blending CO, COM, and COT in various ratios. Table 2 shows the detailed blending ratio and the designation of the PU foams produced therefrom.

### 2.4. Characterization

The FT-IR spectra of the polyol samples were recorded on a KBr disc at a resolution of 4 cm^−1^ over the wavenumber range 4000–500 cm^−1^ using a Nicolet 760 MAGNa–IR spectrometer. A Varian VNMRS 600 MHz spectrometer was used to record the ^1^H-NMR spectra of the polyol samples in CDCl_3_ solvent. To determine the OH value of the polyols, a modified ASTM D1957–86 method was used. The rheological behaviors were measured using an Advanced Rheometer 550 (AR–550, TA Instruments, Dallas, TX, USA) fitted with two parallel plates. Frequency sweep tests were conducted at room temperature within a frequency range of 0.1–100 Hz and a strain of 0.1%. The morphologies of the PU foams were examined using JEOL JSM-6340F (Hitachi Co., Tokyo, Japan) FE–SEM. Apparent density was measured according to the ASTM-D1622 standard. The compressive strength was measured using an Instron 5966 UTM at a rate of 2 mm/min according to the ASTM 1621 standard. The TGA of the PU foams was performed from 30 to 800 °C while heating at 10 °C/min in a N_2_ gas-purged atmosphere.

## 3. Result and Discussion

### 3.1. Characterization of Polyols

**Scheme 1:**
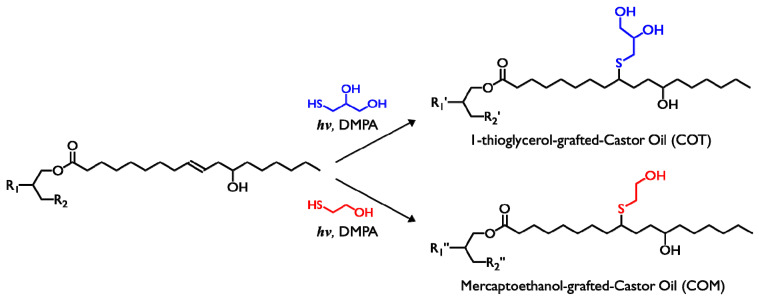
Preparation of castor oil-based multifunctional polyols by the thiol-ene photo-click reaction.

The preparation of castor oil-based polyols via the thiol-ene photo-click reaction is shown in Scheme 1. Based on the optimum reaction conditions reported in our previous research [26], a few modifications were incorporated to produce a large amount of thiol-grafted polyols for PU foam fabrication. The DMPA used in this study was a conventional cleavage type photoinitiator that generates free radicals under UV light. The CO-based multifunctional polyols, COM and COT, were synthesized through a facile and green pathway whose reactions were carried out at room temperature. Theoretically, the number of the primary hydroxyl groups of COT is double that of COM.

**Figure 1:**
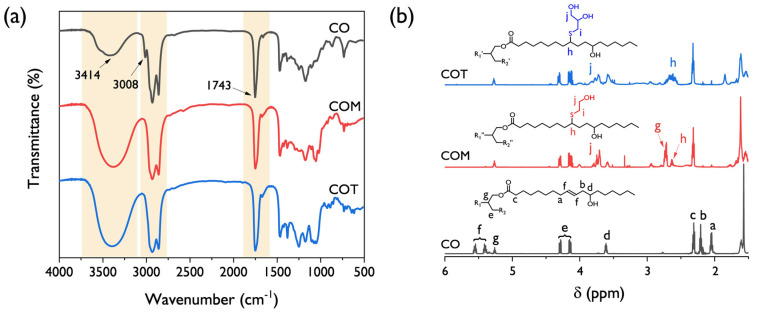
(**a**) FT-IR spectra and (**b**) ^1^H-NMR of CO, COM, and COT.

To characterize the structure of the polyols, FT-IR analysis was carried out. Figure 1a shows the FT-IR spectra of castor oil, COM, and COT polyols. The bands at 1743 cm^−1^ corresponding to the C=O stretching vibrations were used for normalization. The C–S stretching vibration peak in the wavenumber region 800–600 cm^−1^ was too weak. Due to low absorption and positional variability, the bands were not suitable for use in the structural analysis [18]. The grafting of the thiols onto CO resulted in the disappearance of the C=C–H absorption band at 3008 cm^−1^. Due to the presence of the hydroxyl groups of CO, a broad band at 3414 cm^−1^ was observed. For COM and COT, the absorption of the broad bands at 3414 cm^−1^ increased.

The ^1^H-NMR spectra of the polyols are shown in Figure 1b. The peaks shown at δ = 4.1–4.4 ppm (peak e) in all the polyols are attributed to –CH_2_–CH–CH– bonds and were used for normalization. After the thiol-ene photo-click reaction, the peaks between δ = 5.3–5.6 (peak f), corresponding to the C=C bonds in castor oil, completely disappeared. In the ^1^H-NMR spectra of COM and COT, the newly formed peaks at δ = 2.6 and 2.7–2.8 ppm (peak h and i) correspond to the protons on the methylene adjacent to sulfur. Furthermore, the peaks at δ = 3.6–3.8 ppm (peak j) are related to the methylene next to the primary alcohol in the grafted thiols. These observations should be a confirmation of the successful reaction between thiols and castor oil.

The hydroxyl values of CO, COM, and COT were analyzed according to the ASTM D1957–86 standard and the measured values were 152, 259, and 366 mg KOH/g, respectively, which correspond to the values in our previous report [26]. These results confirmed that the synthesis of adequate modified polyols was required for the preparation of PU foams was successful.

### 3.2. Properties of Polyol Blends

**Figure 2:**
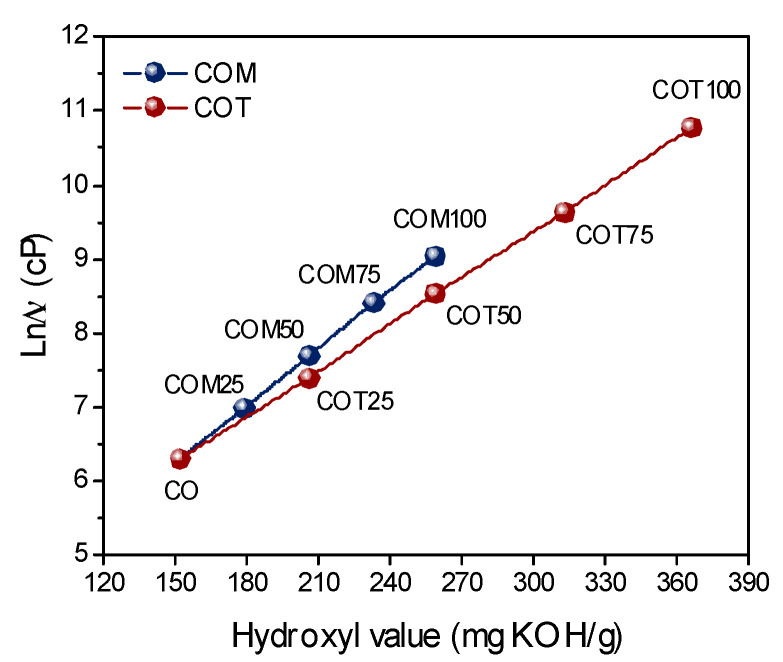
Viscosity as a function of hydroxyl value of the polyol blends at room temperature.

The viscosities of the polyol blends at room temperature are shown in Figure 2. In our previous study, we revealed that the viscosity and activation energy increased significantly with the grafting of hydroxyl groups onto CO. In this study, the viscosities of polyols increased in the order CO < COM < COT, which agrees with our previous results [26]. To analyze the effect of blending COM and COT on the viscosity of the polyols, the viscosities of the polyols were plotted as a function of the OH value. A linear increase in viscosity was observed as the COM and COT contents increased. When comparing COM and COT blends with similar OH values (e.g., COM50 and COT25), a higher viscosity was measured in the COM blends. This can be explained by the fact that the COT blend with a low multifunctional polyol content has a lower viscosity due to the abundance of hydroxyl groups in the molecule, allowing the formation of intramolecular hydrogen bonding.

### 3.3. Preparation of PU Foams

The PU foams were fabricated using CO-based polyols according to the formulation in Table 1. The blending ratios of the polyols are presented in Table 2, as mentioned above. Excess isocyanate was added to react with the blowing agent to generate CO_2_. For comparison, the fabrication of PU foams composed of 100% COM and 100% COT was attempted; however, the PU foams were not formed successfully. The successful fabrication of a PU foam composed of COT75 with a higher OH value and viscosity than PU foam composed of COM100 indicates that the structural characteristics of the polyol have a significant influence on PU foam formation. Next, the morphological, mechanical, and thermal properties of the fabricated PU foams were investigated.

### 3.4. Structural Analysis of the PU foams

**Figure 3:**
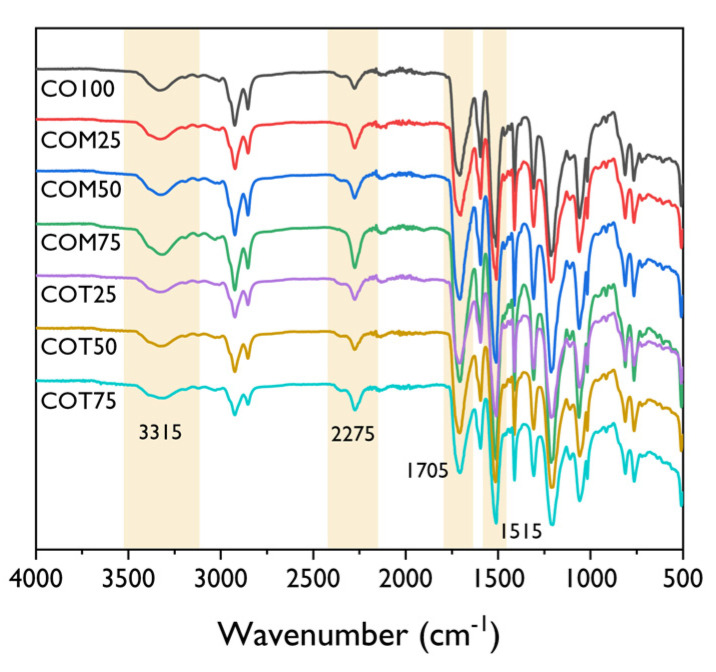
FT-IR spectra of the obtained PU foams.

The FT-IR spectra of the PU foams are shown in Figure 3. The absorption bands at 3315 and 1705 cm^−1^, which correspond to the N–H stretching and C=O carbonyl stretching, respectively, confirm that the urethane linkage is formed successfully. Two split peaks at 2925 and 2853 cm^−1^ are attributed to symmetric *sp*^2^ and asymmetric *sp*^3^ stretching bands in the aliphatic chain of CO. The bands observed at 1212 and 1052 cm^−1^ correspond to the C–O–C antisymmetric and symmetric stretching vibrations, respectively. There was no significant change in the absorption band by using COM or COT polyol blends. However, the distinctive peaks detected at 2275 cm^−1^, which correspond to the N=C=O stretching vibration, demonstrate that unreacted NCO groups remained in all the synthesized PU foams. This was expected, as a 20% excess of NCO was added for CO_2_ release [32,33]. In the cases of COM50 and COT50, the intensity of the isocyanate peaks decreased, whereas for COM75 and COT75, the ratio of unreacted NCO appeared to increase. These observations indicate that the composition of the polyol blend affected the formation of the PU foam structure.

### 3.5. Morphology

**Figure 4:**
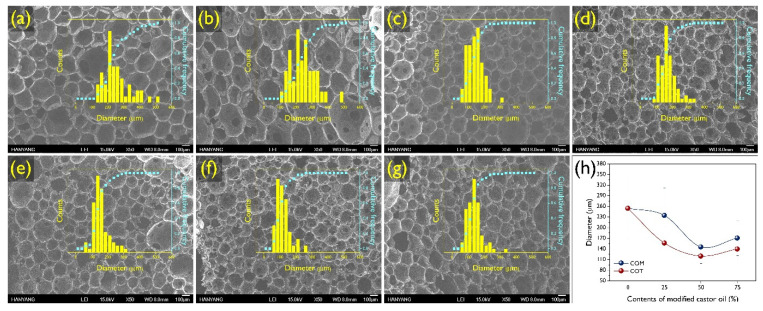
SEM micrographs and cell diameter distribution of PU foams prepared with various polyols: (**a**) CO, (**b**) COM25, (**c**) COM50, (**d**) COM75, (**e**) COT25, (**f**) COT50, and (**g**) COT75. (**h**) Changes in the average cell diameter of the PU foams.

The cellular morphologies of the COM- and COT-based PU foams are illustrated in Figure 4. The average cell diameter was calculated from the diameter estimates of 100 cells identified in SEM images. The cells of the PU foams fabricated using CO or polyol blends were mostly composed of closed cells. CO100 (Figure 4a) showed the largest average cell diameter of 254 μm. As shown in Figure 4b–d, the average cell size decreased as the COM content increased up to 50%, however, a larger cell size was observed at a higher COM ratio. A similar trend was also observed in PU foams composed of COT. The average cell sizes of COM50 and COT50 were 146 and 119 μm, respectively. Considering that COM100 and COT100 were not formed successfully, one can assume that the content of the multifunctional polyol had a significant influence on the PU foam formation. In general, the low viscosity of the polyol leads to the formation of a larger cell size because it can be easily merged with adjacent cells due to delayed crosslinking of the PU foam wall. The cell size decreased gradually and uniform distribution appeared as the content of COM or COT increased up to 50%. On the other hand, at very high viscosities, the growth of carbon dioxide bubbles may be hindered by uneven and rapid crosslinking, resulting in an uneven size of cells in the PU foams. The cell size of PU foam can be affected by a complex interaction of various factors, including polymerization rate, hard block segregation, and crosslinking density [34]. In particular, the crosslinking density determines the strength of the cell wall of PU foam [35]. The increase in cell size and unevenness for COM75 and COT75 can be explained by the formation of weak cell walls due to the increase in unreacted hydroxyl groups during the rapid foaming process. Moreover, the reason why the COM100 and COT100 could not be obtained can be conjectured to be that they did not have sufficient strength to maintain the foam structure. It can be assumed that the excess amount of branched polyols cause steric hindrance to crosslinking with NCO and low intermolecular forces, reducing the strength of the generated PU. The presence of unreacted NCO groups observed in the FT-IR section gives evidence for the formation of weak cell walls. Thus, it can be assumed that blending COM or COT with the appropriate proportion of CO can lead to a successful PU foam formation. The result of the morphological analysis supports this assumption.

### 3.6. Density

**Figure 5:**
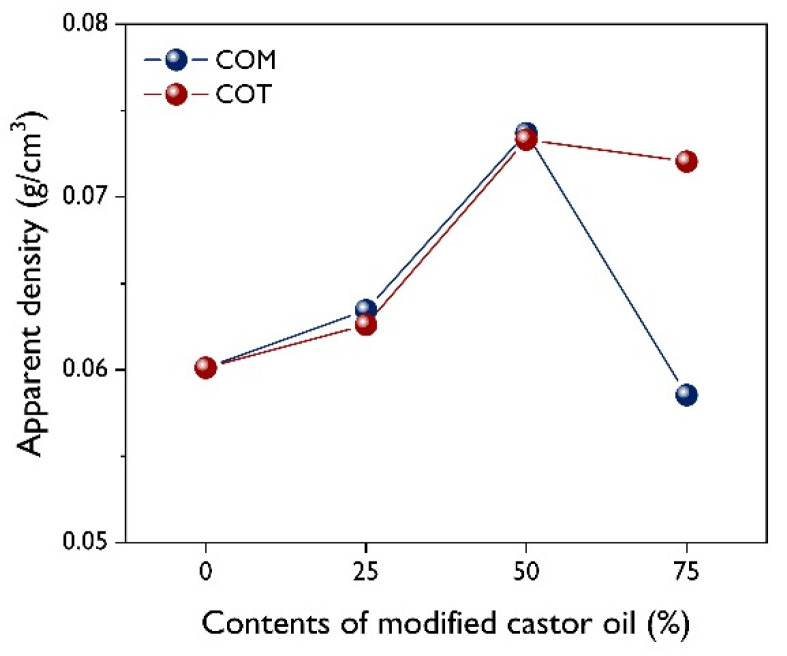
Effect of modified castor oil contents on the density of obtained PU foams.

Figure 5 shows the apparent densities of CO-based PU foams with various COM or COT contents. The apparent density of PU foams increased from 0.060 to 0.073 g/cm^3^ as the COM content increased from 0 to 50%. Even for the COT blend, the apparent density of the PU foams increased up to 0.073 g/cm^3^. As previously stated, upon increasing the COM or COT content, the viscosity of the polyol blends increases, which led to an increase in the apparent density of the PU foams. This is because the increased polyol viscosities limit expansion during foam formation [36]. However, for COM75 and COT75, a decrease in apparent density was observed. From the results of the FT-IR and morphological analyses discussed earlier, it is presumed that the amount of unreacted NCO and number of OH groups increase as the content of modified polyol increases, resulting in a decrease in the density of the PU itself. Despite of the increase in the ratio of modified polyols, it is presumed that the increase in unreacted NCO is because of the structural bulkiness of the modified polyol. The addition of an adequate amount of multifunctional branched polyol improves the reactivity and promotes PU synthesis, however, an excessive amount of modified polyol hinders the sufficient formation of cell walls, leading to the merging of cells and a reduction in density. Moreover, unreacted NCO groups promote foam rising, resulting in larger cell size and lower density. In the case of COT75, owing to the abundance of pendant groups prevalent in the CO backbone, which can act as nucleation sites, the rate of the decrease in density was relatively low.

### 3.7. Mechanical Properties

**Figure 6:**
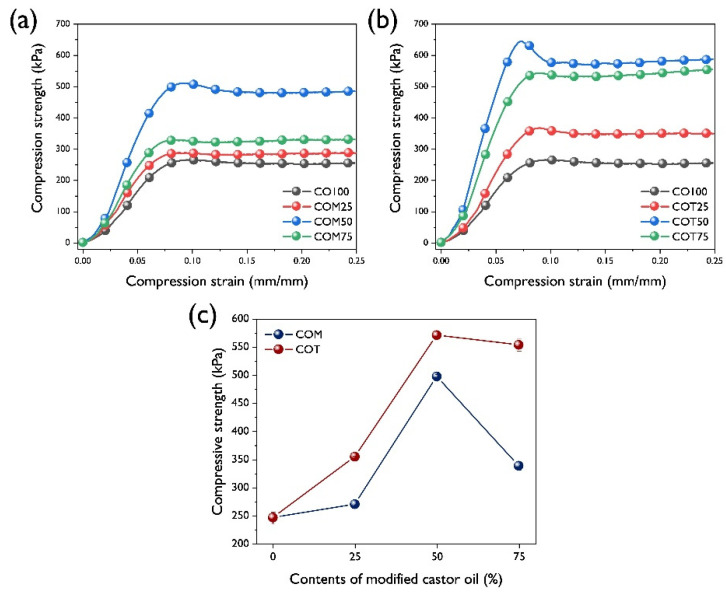
Compressive stress–strain curves of the PU foams prepared from (**a**) COM and (**b**) COT. (**c**) Compressive yield stress of the PU foams made from COM and COT.

The compressive stress–strain curves of the PU foams fabricated from various polyol blends of COM and COT are shown in Figure 6a,b. Initially, a linear elastic deformation was observed owing to the viscoelastic response of the PU foams. This was followed by a plateau of deformation [34,37]. For all PU foams, compressive stress increased with increasing compressive strain. The mechanical properties of the PU foams are affected by various factors, such as cell morphology, crosslinking density, and apparent density [7]. As the blending ratio of COM and COT increased, the compressive strength of the PU foams significantly increased from 246 (CO) to 497 (COM50) and 571 (COT50) kPa. This was attributed to the finer cell structure of the PU foams and the higher viscosity of the polyol blends that led to improvements in mechanical properties. For COM75 and COT75, the compressive strength decreased because of the decrease in the density. Based on the higher compressive strength of the PU foams fabricated from COT polyol blends compared to the PU foams fabricated from COM polyol blends of similar apparent density, we assumed that the PU foams fabricated from COT polyol blends were formed with a higher crosslinking density due to their higher viscosity and OH value of the COT blends.

### 3.8. Thermal Stability

**Figure 7:**
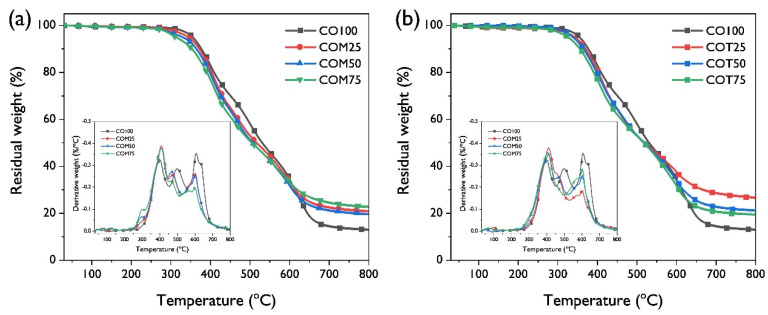
TGA thermograms and their derivative curves (inset) of the obtained PU foams using (**a**) COM and (**b**) COT.

To estimate the thermal stability of the PU foams fabricated from COM and COT polyol blends, TGA was performed under a nitrogen atmosphere. The TGA thermograms and their derivative curves are shown in Figure 7. The thermal decomposition parameters based on many previous studies revealed that the thermal degradation behavior of vegetable oil-based PU occurs in three steps [38]. First, the urethane bonds, which have relatively poor thermal stability, begin to decompose. During this step, three degradation mechanisms of the urethane bonds occur simultaneously, namely, the dissociation into NCO and alcohol, the formation of primary amines and olefins, and the formation of secondary amines and dioxides. The lowest decomposition temperature during this stage was 401 °C for the PU foam synthesized from CO and the highest was 413 °C for COT50. The second and third degradation steps occurred at similar temperatures for all PU foams regardless of the type of polyol blend. Steps two and three are associated with the oligomerization of the triglyceride structure in CO and the degradation of the remainder of the second step, respectively. The distinctive difference in the thermal degradation temperature mainly occurs in the first step depending on the polyol blends. Hence, it can be assumed that the decomposition of the carbon–sulfur bond occurs in the first step.

### 3.9. General Discussion

PU foams were successfully fabricated from multifunctional CO-based polyols via a thiol-ene click reaction. Both the structural characteristics of the polyols and the composition of the polyol blend affected the characteristics of the PU foam. By blending COM or COT with an appropriate proportion of CO, an increased viscosity of the polyol blend was achieved, which led to an increase in the density of the PU foam.

Compared to the PU foams fabricated from COM polyol blends, the PU foams fabricated from COT polyol blends were formed with a higher crosslinking density, which resulted in higher compressive strength.

## 4. Conclusions

CO-based multifunctional polyols were synthesized via a facile thiol-ene click reaction. Structural analyses of the prepared polyols were performed by FT-IR and ^1^H-NMR spectroscopy. The results demonstrated that the thiol-ene click reaction used in this study is a scalable method, which can enable the mass production of thiol-grafted polyols. Polyol blends were prepared by mixing CO with COM and COT, and their viscosities were analyzed. PU foams were fabricated using polyol blends, and their structures were determined via FT-IR analysis. It was confirmed that the PU foam structure was affected by the composition and ratio of the blended polyols. Morphological analysis of the PU foams revealed that an optimal proportion of CO in the polyol blends led to the formation of a dense structure. As the blending ratios of COM and COT increased up to 50%, the compressive strengths of the PU foams increased by 50 and 75%, respectively. Overall, we believe that bio-based PU foams made of a CO-based multifunctional polyol may find a wide range of applications that require high compressive strength.

## Figures and Tables

**Table 1:** Formulation of the polyurethane (PU) foam.

Materials		Formulation (php ^a^)
Polyol		100
Polymeric diphenylmethane diisocyanate	NCO index	120
Chain extender	1, 3-Propanediol	10
Silicon surfactant	TEGOSTAB B-4862	2
Catalyst	DMCHA	0.2
	DBTDL	0.1
Blowing agent	Water	3

^a^ php: parts per hundred of polyol by weight.

**Table 2:** Designation of synthesized PU foams and composition of polyol blends.

PU Foam	CO (%)	COM (%)	COT (%)	OH Value
CO100	100	-	-	152
COM25	75	25	-	179
COM50	50	50	-	206
COM75	25	75	-	233
COM100	-	100	-	259
COT25	75	-	25	206
COT50	50	-	50	259
COT75	25	-	75	313
COT100	-	-	100	366

CO: Castor Oil, COM: CO-based polyols modified with mercaptoethanol, COT: CO-based polyols modified with α-thioglycerol.

## Data Availability

The data presented in this study are available on request from the corresponding author.
